# A Critical Review of Phenolic Compounds Extracted from the Bark of Woody Vascular Plants and Their Potential Biological Activity

**DOI:** 10.3390/molecules24061182

**Published:** 2019-03-26

**Authors:** Corneliu Tanase, Sanda Coșarcă, Daniela-Lucia Muntean

**Affiliations:** Faculty of Pharmacy, University of Medicine, Pharmacy, Sciences and Technology of Târgu-Mureș, Gh. Marinescu Street No. 38, RO-540139 Tîrgu Mureș, Romania; sandacosarca31@gmail.com (S.C.); danielaluciamuntean@yahoo.com (D.-L.M.)

**Keywords:** bark, biological properties, extraction, polyphenols, rhytidome, woody vascular plants

## Abstract

Polyphenols are one of the largest and most widespread groups of secondary metabolites in the plants world. These compounds are of particular interest due to their occurrence and the properties they possess. The main sources of phenolic compounds are fruits and vegetables, but lately, more and more studies refer to woody vascular plants, especially to bark, as an important source of phenolic compounds with a potential biological effect. This study aims to bring together information on the phenolic compounds present in the bark of woody vascular plants by discussing extraction methods, the chemical composition of the extracts and potential biological effects. The literature data used in this paper were collected via PubMed (2004–2019). Search terms were: bark, rhytidome, woody vascular plant, polyphenols, phenolic compounds, biologic activity, antioxidant, immunostimulatory, antimutagenic, antibacterial, anti-inflammatory, and antitumoral. This paper intends to highlight the fact that the polyphenolic extracts obtained from the bark of woody vascular plants represent sources of bioactive compounds with antioxidant, immunostimulatory, antimutagenic, antibacterial properties, etc. Future research directions should be directed towards identification and isolation of bioactive compounds. Consequently, biologically active compounds obtained from the bark of woody plants could be exploited on an industrial scale.

## 1. Introduction

Current research is directed towards finding new sources of biologically active natural compounds with a wide range of applicability. Polyphenolic compounds are of particular interest due to their occurrence and properties. Phenolic compounds or polyphenols are one of the most frequent and widespread groups of substances in the world of plants, with more than 8000 identified phenolic structures [1]. These compounds can be found in almost all organs of a plant, and according to their structure, they have different functions ranging from skeletal constituents of different tissues to pigmentation of several plant organs [2]. Polyphenols are secondary metabolites essential for the growth and development of plants and their reproduction. Similarly, they help to control growth in diameter, pigmentation, and defence against various pathogens [3] or act as signalling molecules to distinguish symbionts [4]. These compounds, as natural antioxidants, have important properties that involve the inhibition of lipid peroxidation, inhibition of carcinogenesis, antimicrobial activity, direct constrictive action on capillaries, naturally occurring phytohormones, stabilisation of ascorbic acid, etc. [5].

The present paper is a critical review of the literature (2004–2019) on extraction methods of phenolic compounds from the bark of woody vascular plants, and their chemical composition, with an emphasis of their potential biological properties.

## 2. The Bark of Woody Vascular Plants—Source of Phenolic Compounds

The bark or rhytidome is a set of dead tissues, developed after the primary and secondary growth of bark (multiple layers of periderms), which together form the protective layers of branches and the trunks of woody vascular plants. The bark inhibits water loss through evaporation, has a protective role against overheating, frost, herbivores or infestation with parasites. The bark comprises up to 20% of the dry weight of woody vascular plants and contains polysaccharides, lignin, suberin, suberan, tannins or phenolic acids [6].

Currently, the woodworking industry produces a large amount of residue each year as a result of debarking woody vascular plants. Commonly, huge amounts of bark of woody plants can be found among wood wastes in the forest. These wastes are usually used for heating or as a cheap source of energy in cellulose factories, although these kinds of exploitations are not efficient and can lead to environmental problems [6]. 

**Figure 1:**
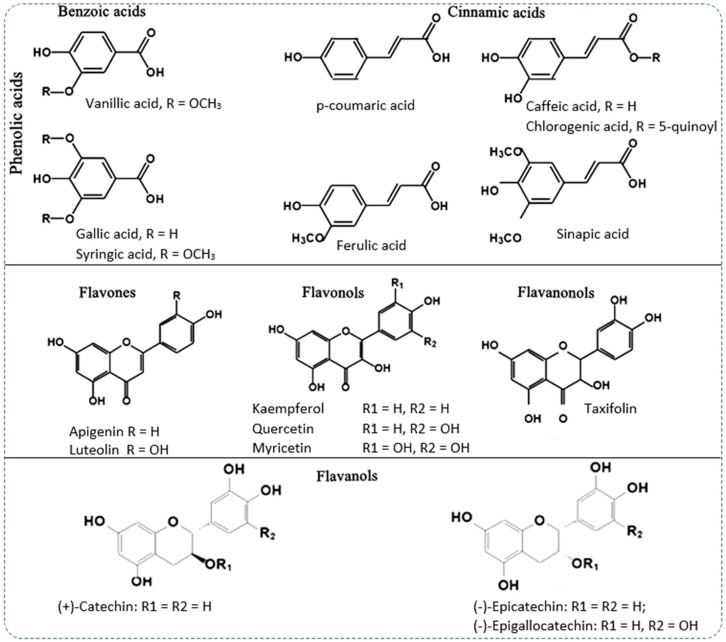
Typical phenolic compounds identified in the bark of woody vascular plants.

The main sources of phenolic compounds are fruits and vegetables, but more studies refer to woody vascular plants, especially to bark, as an important source of phenolic compounds with a potential biological effect. Polyphenols, according to their chemical structure, are divided into sub-groups (Figure 1): phenolic acids (hydroxybenzoic and hydroxycinnamic acids), flavonoids (flavonols, flavones, flavanones, flavanonols, isoflavones, anthocyanidins, tannins), stilbenes (resveratrol) and lignans found in plants and foods of plant origin [7,8].

Phenolic acids are one of the main classes of phenolic compounds found in plants and occur in the form of esters, glycosides or amides, but rarely in free form. The structural variation of phenolic acids depends on the number and position of hydroxyl groups on the aromatic ring. Phenolic acids have two distinctive structures: the hydroxycinnamic and hydroxybenzoic acid (Figure 1). The most common benzoic acids found in the bark of woody plants are vanillic, gallic, syringic and protocatechuic acid [9,10,11]. The most common cinnamic acids are *p*-coumaric, caffeic, ferulic and synaptic acid [12,13].

Flavonoids are composed of two aromatic rings linked by a unit of three carbon atoms (C6-C3-C6). This carbon skeleton is the explanation for the chemical diversity of this family of compounds. The basic structures of flavonoids are aglycones but in plants, most of these are as glycosides [1]. The most common sub-groups of flavonoids found in bark of woody plants are flavonols (quercetin, kaempferol, myricetin, etc) [11,14,15,16,17], flavanonols (taxifolin), [14,16,18] flavones (apigenin, luteolin) flavanols [19] (catechin, epicatechin) [10,14,16,20] and tannins [18,19].

Stilbenes are phenolic compounds that contain two aromatic rings connected by a heterologous bridge. Resveratrol (3,5,4′-trihydroxystilbene) is the reference stilbene in grapes and wine [21] but it was identified in barks of *Picea mariana* (Mill.) Britton, Pinaceae [11] or *Malus domestica* Borkh, Rosaceae [22].

Lignans are dimers of phenylpropanoids, which result from the tail-to-tail binding of two coniferyl or sinapyl alcohol units. Examples of such compounds include isolariciresinol, secoisolariciresinol, lariciresinol, cedrusin and their glycosides [23], which present increasing interest in lignans especially due to their chemotherapeutic potential [24].

Bark contains large quantities of phenolic compounds and lignin. Thus, it can be considered as a possible renewable source of bioactive compounds, especially of aromatic substances. For example, Hofmann et al. [25] studied beech (*Fagus sylvatica* L., Fagaceae) bark and determined that the total polyphenol content was approximately 57 mg gallic acid (GAE)/g dry bark units. The most efficient compounds with potential antioxidant activity in beech bark are epicatechin, coumaric acid, coniferin, quercetin, taxifolin-*O*-hexoside, coumaric acid-di-*O*-hexoside, syringic acid-di-*O*-hexoside, coniferyl alcohol-*O*-hexoside [26].

Another source of woody plant rich in phenolic compounds is the bark of black poplar (*Populus nigra* L., Salicaceae) with a total polyphenol content between 96.69–334.87 mg GAE/g dry bark units [27]. The bark of *Schinopsis brasiliensis* Engl., Anacardiaceae, is also an important source of polyphenols. The most important phenolic component identified as a chemical marker of *S. brasiliensis* is gallic acid [28].

## 3. Methods Used to Extract Phenolic Compounds from the Bark of Woody Vascular Plants

The chemical composition of a plant product is determined by qualitative chemical analysis using various solvents for extraction. The choice of method and solvent used for extraction is a particularly important step to obtain an optimal concentration of natural compounds in the extract. It is important to select an efficient extraction method and proper work phases to assure high performance and increased stability of the extracted compounds [2].

The most commonly applied methods for the extraction of polyphenols use water in combination with organic solvents (acetone, ethanol, methanol, ethyl acetate) as per the type of polyphenols present in the bark of the plant [29]. Several authors reported increase of the extraction temperature could be correlated with increased efficiency [16]. Extraction time is a factor that should be taken into consideration as well. Prolonged extraction time can influence the oxidation process of polyphenols thus possibly decreasing the efficiency of the procedure and the type of extracted compounds [30].

Solid-liquid extraction is a common method used for obtaining polyphenols [31,32]; however, there may be various shortcomings such as long extraction time, increased quantity of solvent use, reduced potential to recover the solvent, which implies higher costs and higher toxicity. To improve extraction yield, time, and used solvent quantity, some unconventional (modern) methods such as ultrasonic extraction, microwave-assisted extraction, supercritical fluid extraction, pressurised liquid extraction or accelerated solvent extraction are preferred. The advantages of these methods compared to conventional methods (classical water bath extraction, Soxhlet extraction, and maceration) are the reduction of extraction time and quantity of required extraction solvent, as well as high reproducibility [33].

Ultrasound extraction is an effective alternative of conventional extraction methods, and the main advantage is its simplicity, the required equipment and the reduced extraction time [33,34]. However, in comparison with other modern methods, this one uses the highest amount of solvent and has the longest extraction time [33]. On the other hand, Chen et al. [35] demonstrated that ultrasonic extraction of the bark of *Betula papyfera* Marshall, Betulaceae, by using ethanol and water as a solvent has a maximum extraction yield at 180 min and 50 °C. The optimal conditions for ultrasound extraction of polyphenols from the bark of *Eucalyptus camaldulensis* Dehnh., Myrtaceae, and *Flourensia cernua* DC., Asteraceae, are at 40–50 °C by using ethanol as extraction solvent [36].

During microwave extraction, the solid matter and solvent are subjected to microwave treatment, which accelerates the process of extraction due to the heating of the system. Thus, water within the vegetal matrix absorbs microwaves, and cell disruption occurs through internal superheating, which facilitates desorption of extractives from the vegetal matrix. This method uses polar solvent or a mixture of miscible polar solvents, because non-polar solvents do not or barely absorb microwave radiation. The advantages of this method lie in the fact that extraction time and the quantity of the solvent are reduced, whereas the efficiency of the extraction method is improved in comparison with conventional extraction methods [33]. Compared to other methods of polyphenol extraction, microwave-assisted extraction has proved to be efficient because of its shorter processing time [37]. It was observed that during the process of microwave extraction, time and microwave power are the main factors that influence efficiency significantly. It has also been noticed that the combination of miscible polar solvents improves the extraction yield [22,26,38,39]. 

Supercritical fluid extraction is an alternative solid-liquid extraction where the extraction solvent is replaced with a supercritical fluid (most commonly with carbon dioxide, but also with other materials such as nitric oxide, ethane, propane, n-pentane, ammonia, and water). This is a relatively new method of processing solid and semi-solid substances, which has since become a specific technique referred to as supercritical fluid chromatography. The most important property of supercritical fluids during the extraction process is the ability to adjust solubility through physical parameters such as temperature and pressure, so that a supercritical fluid can extract a group of analytes of different polarities and molar masses in a more or less restricted fashion, and to reduce the volume of solvents used during extraction [40,41]. Supercritical fluid extraction was used to extract polyphenols from the bark of *Hymenaea coubaril* L., Fabaceae, by using CO_2_, CO_2_ + ethanol and CO_2_ + water as solvents, with the highest extraction yield being achieved with the combination of CO_2_ + water [42].

Accelerated solvent extraction is a new extraction method based on the use of high temperature and pressure to accelerate dissolution kinetics and to break the bonds of analyte-matrix interaction. Hence, this method is also referred to as pressurised fluid (solvent) extraction [30]. Moreover, by increasing the temperature the viscosity of the solvent decreases, which facilitates penetration of the solid matrix. This way, extraction time is reduced from tens of minutes to a couple of minutes, and extraction samples can be processed in small quantities. This method is an alternative of the Soxhlet or supercritical fluid extraction techniques [43]. 

Numerous studies (Table 1) have focussed on optimising methods of extracting polyphenols from the bark of woody vascular plants. In addition to conventional extraction methods, modern methods of polyphenol extraction are widely used as well [27,39,44,45,46]. Thus, for the same studied species, different values of total phenolic contents (TPC) can be obtained. For example, it was observed that for the *Eucalyptus* species the Soxhlet extraction method was preferred, thus obtaining a higher extraction yield of the total phenol content [47].

It has also been remarked that extraction temperature influences TPC and the type of extracted compounds. For example, *Populus nigra* L., Salicaceae, extracts obtained at temperatures above 200 °C displayed a higher content of flavonoid secondary metabolites and other polyphenols, and the level of antioxidant activity was higher than in the extracts obtained at temperatures below 180 °C [27]. 

Paz et al. [36] started researching four woody vascular plants *Eucalyptus camaldulensis* Dehnh., Myrtaceae, *Flourensia cernua* DC., Asteraceae, *Jatropha dioica* Sessé, Euphorbiaceae, and *Turnera diffusa* Willd. ex Schult., Passifloraceae. They used the ultrasound-assisted technique to obtain optimised extracts by adjusting the extraction time and solvent concentration (ethanol). Optimal conditions for extraction were created at 40 min of extraction time and 35% ethanol concentration. 

Another intensively studied potential source of polyphenols is the bark of *Picea abies* L., Pinaceae. Researchers have attempted to optimize different methods of polyphenol extraction by changing the temperature, solid-liquid contact surface and extraction time in the presence of ultrasounds [48,49] and classical water bath extraction techniques [19,50]. For example, Lazar et al. [49] concluded that ultrasounds and temperature lead to significant effects on the polyphenolic compounds from spruce bark. Thus, the total content of polyphenols increased from 37.3 mg GAE g^−1^ / spruce bark / 45 °C to 43.1 mg GAE g^−1^ / spruce bark / 60 °C [49].

In a study on the bark of *Ulmus pumila* L., Ulmaceae, conducted by Zhou et al. [51], the highest extraction yield was observed in the case of enzyme-assisted extraction (enzyme mixtures including cellulase, pectinase, and β-glucosidase) at pH = 4.63, 52.6 °C and 62 min when the total polyphenol content was 16.04 mg gallic acid/g dry matter. 

Recent studies have highlighted the bark extracts of *Terminalia arjuna* (Roxb.) Wight & Arn., Combretaceae, obtained with the use of various organic solvents, the highest polyphenol content extracted with butanol. The use of chloroform proved to have the lowest extraction capacity [52].

Hofmann et al. [25] aimed to optimise extraction methods according to the duration of exposure to ultrasounds and microwaves, solvents concentration and temperature. Thus, it was observed that the largest amount of polyphenols was obtained when the microwave-assisted extraction (MAE) technique was applied for 20 min by using ethanol and water as solvents. The extract with the most prominent antioxidant activity was obtained by the conventional extraction technique using water as solvent [25].

Vásquez et al. [14] identified significant amounts of polyphenols in the bark of *Eucalyptus globulus* Labill., Myrtaceae and *Castanea sativa* Mill., Fagaceae. They performed extractions using different solvents in different amounts. Regarding the total polyphenol content, the best extraction yield was obtained by using the conventional methanol-water extraction method for both the bark of *E. globulus* (TPC = 20.1 g GAE/100 g extract) and the bark of *C. sativa* (TPC = 59.7 g GAE/100 g extract), the only difference being the solvent ratio. They also determined the antioxidant activity (AOA) of the extracts obtained with different solvents and noticed that in the case of the bark of *C. sativa*, the extract with the best AOA was extracted with a solution of 2.5% sodium bisulfite, and for the bark of *E. globulus*, the methanol: water extraction type (50:50, *v*/*v*) provided the best results.

Woody plant extracts like *Jatropha dioica* Sessé, *Fluorensia cernua* DC., *Turnera diffusa* Willd. ex Schult. and *Eucalyptus camaldulensis* Dehnh. studied by Paz et al. [36], showed the highest value of total polyphenol content at 40 min extraction time and 35% ethanol concentration.

In a study regarding the composition and extraction yield of phenolic compounds of the species *Acer saccharum* Marshall, Sapindaceae and *Betula alleghaniensis* Britt., Betulaceae, we could observe similarities in the case of both ultrasound and maceration extraction, with the total polyphenol content being similar in the two species [56]. The bark of *B. alleghaniensis* was also studied by Diouf et al. [93] when the same extraction methods were used to determine the TPC and to identify triterpenic compounds such as lupenone, lupeol, betulinic acid, betulone, betulin and acetyl methyl betulinate.

In 2015, Enkhtaivan et al. [61] performed a comprehensive study on the total content of polyphenols in the bark of the following species *Cayratia pedata* (Lam.) Gagnep, Vitaceae, *Chloroxylon swietenia* DC., Rutaceae, *Diotacanthus albiflorus* Benth., Acanthaceae, *Strychnos minor* Dennst., Loganiaceae and *Strychnos nux-vomica* Dop., Loganiaceae. The results showed that the bark of *D. albiflorus* had the highest content of polyphenols (29.73 mg GAE/g dry plant material). The polyphenol content of *D. albiflorus* and *Strychnos nux-vomica* was higher in their bark than in their leaves.

The phytochemical analysis of the bark of *Acacia ferruginea* DC., Myrtaceae, extract showed the presence of alkaloids, flavonoids, triterpene, tannins and the total polyphenolic content was about 438 mg GAE/g dry plant material [55].

## 4. Biological Effects of Extracts Obtained from the Bark of Woody Vascular Plants

Phenolic compounds are known for their role in regulating the immune system, their anti-inflammatory effect, chemoprevention, neuroprotection, cardioprotection and in the treatment of diseases such as diabetes, Parkinson’s disease and cancer; in addition to this, they also have antibacterial [58,94] and antivirals effects [61]. Furthermore, the potential biological effects of some polyphenolic extracts obtained from the bark of woody vascular plants are presented (Table 2).

### 4.1. Antioxidant Effect

Polyphenols are compounds with one or more hydroxyl groups attached to the benzene ring. This structural feature provides a stronger acidic character to phenol than does to other alcohol groups. This chemical reactivity is responsible for the antioxidant character of polyphenols. The ability of polyphenols to capture free radicals is largely dependent on the number of hydroxyl groups [14,84,87,95]. There is a strong correlation between total polyphenol contents and antioxidant activity [90]. The main components possibly responsible for the antioxidant character of the *T. arjuna* extract were identified to be gallic acid, apigenin, luteolin, quercetin, epicatechin, ellagic acid [52]. In 2012, Santos et al. [47] studied three species of *Eucalyptus*, Myrtaceae, namely *E. grandis* W.Hill ex. Maiden, *E. maidenii* F. Muell and *E.* x *urograndis*. These species proved to have higher antioxidant potential than *E. globulus* [32]. The bark of *E.* x *urograndis* was found to have the highest antioxidant activity (IC50 = 8.24 μg mL^−1^) and the best extraction yield (15.18%) compared to the other species included in the study (10.54% for *E. grandis* and 13.23% for *E. maidenii*). Thus, the potential antioxidant effect of some polyphenolic global extracts obtained from the bark of woody vascular plants are presented in Table 2.

### 4.2. Anti-Inflammatory Effect

It has been demonstrated that besides their antioxidant effect, polyphenols reduce lipid peroxidation and DNA damage [96,97,98]. They also trigger a mechanism that blocks the overproduction of the tumour necrosis factor (TNF-α), thus exerting an anti-inflammatory effect [27]. 

An increase in nitric oxide (NO) synthesis was observed in the inflamed tissues, and quercetin appeared to reduce the synthesis of nitrogen monoxide by inhibition of NO synthase [99]. For example, the *Allophylus africanus* P. Beauv., Sapindaceae, extract presented anti-inflammatory effects, successfully inhibiting the enzyme involved in the mechanism of inflammation, namely 5-lipoxygenase, which would be explained by the high quantity of flavons in the extract [45]. Other polyphenolic extracts obtained from the bark of woody vascular plants, with potential anti-inflammatory effect are presented in Table 2.

### 4.3. Antibacterial Effect

Besides the antioxidant and anti-inflammatory activity of certain phenolic extracts, antimicrobial effects have also been observed. Several studies have been conducted on antibacterial activity. It was established that the ethanolic extract obtained from *Picea abies* L., Pinaceae, has antibacterial activity against Gram-positive (methicillin-resistant *Staphylococcus aureus*) and Gram-negative bacteria (*Pseudomonas aeruginosa* and *Klebsiella pneumoniae*) [100]. The study of the bark extract of *Fagus sylvatica* L., Fagaceae, underlined the antimicrobial activity against methicillin-resistant *Staphylococcus aureus* [101].

The methanolic extracts of some African herbs *Terminalia arjuna* Wight & Arn, Combretaceae, *T. brownie* Fresen., Combretaceae and *Anogeissus leiocarpus* DC., Combretaceae, have revealed antimicrobial effects. In these extracts the combinations of different phenolic compounds such as ellagic acid, gallic acid, and ellagitannins were identified, but when they were separated and tested the antimicrobial effect decreased in comparison with the raw extract [58]. Another study on the antibacterial activity of *T. arjuna* revealed that the extracts of the bark presented the highest antibacterial activity. This effect has been tested against bacteria such as *Bacillus subtilis, Staphylococcus aureus, Escherichia coli, Klebsiella pneumoniae, Pseudomonas aeruginosa* and *Salmonella typhi* [90]. It was observed that the butanolic extract of bark was more effective in bacterial inhibition than extracts using water, chloroform or ethyl acetate as solvent, this being also correlated with higher values of TPC in butanolic extract, with 294 mg/g GAE versus 270 mg/g GAE in chloroform extract, and 189.9 mg/g GAE in aqueous extract.

Other polyphenolic extracts obtained from the bark of woody vascular plants, with potential antibacterial effect are presented in Table 2. The results of these studies open new research directions aimed at reducing pharmacological resistance of microorganisms to antibiotics by using plant phenolics.

### 4.4. Other Effects

Arunachalam and Parimelazhagan [68] researched the effects of the bark of *Ficus talboti* King, Moraceae, extract in diabetic rats with induced pathology. Their results were promising because they noticed that blood levels of triglycerides and cholesterol were reduced, body weight decreased, and the antidiabetic action was comparable to glibenclamide. They also observed that the activity of endogenous enzymes with antioxidant effect and insulin sensitivity of β-pancreatic cells increased. The authors suggested that the antidiabetic effect is due to the presence of routine, quercetin and kaempferol. The alcoholic extract of *F. racemosa* L. was proven to have a higher antioxidant effect than the aqueous extract [68]. 

The bark of *Picea mariana* (Mill.) Britton, Pinaceae, was studied and authors identified numerous phenolic components with important therapeutic action [11]. Thus, the phenolic compounds of lignan, neolignan, phenolic acids, and flavonoid classes were identified with important anti-inflammatory and antiproliferative activity with high potential of capitalisation in the pharmaceutical industry.

*Erythrina suberosa* Roxb., Fabaceae, an ornamental plant in India, has been studied concerning the cytotoxicity in leukaemia cell lines. Thus, it was concluded that 4′-Methoxy licoflavanone (MLF) and Alpinumisoflavone (AIF) inhibit the proliferation of HL-60 cells and induce their apoptosis [64].

Enkhtaivan et al. [61] investigated the bark of certain medicinal plant species like *Cayratia pedata* Lam., *C. swietenia, D. albiflorus, S. minor,* and *S. nux-vomica* L. and found a high antioxidant potential correlated with antiviral activity against H1N1 virus and cytotoxicity in Madin-Darby Canine Kidney (MDCK) cell lines.

*Rhus verniciflua* (Stokes) F. Barkley, Anacardiaceae, is a plant that has neuroprotective and anti-neuroinflammatory potential, and at the same time it enhances cognitive functions by protecting neurons against oxidative stress [102]. The neuroprotective and anti-inflammatory effect was tested in vitro, and the improvement of cognitive functions was highlighted by in vivo studies. The compounds responsible for the above mentioned effects appear to be the flavonoids named fisetin and butein, since fisetin increases the intracellular levels of glutathione and inhibits the activity of cyclooxygenase-2 (COX-2) and type II nitric oxide synthase (iNOS), which makes it an excellent therapeutic candidate for diminishing the progression of Alzheimer’s disease and other neurodegenerative diseases. Another recently published study has highlighted the neuroprotective, antidepressant, anti-inflammatory and antioxidant effect of the aqueous phenolic extract from the bark of *Trichilla catigua* A. Juss., Meliaceae. The extract contained predominantly quinic acid derivatives, flavan-3-ols and phenylpropanoid substituted flavan-3-ols, largely responsible for the neuroprotective effect of the plant [91].

The hydroalcoholic extract of the bark of *Acacia ferruginea* DC., Myrtaceae, presents important therapeutic potential, considering that it is rich in flavonoids, triterpenoids, saponins, tannins and alkaloids. Faujdar et al. [55] studied this hydroalcoholic extract and confirmed its anti-inflammatory and anti-hemorrhoidal activity, but it has not been determined exactly which components of the extract have these specific effects.

The bark extract of *T. catigua* has been used empirically in the Brazilian traditional medicine for its neurostimulation and antidepressant effects. Recent studies have validated the traditional use and have demonstrated that the aqueous extract has been considered to have anti-inflammatory, antidepressant and neuroprotective effects due to the flavan-3-ol content and its phenylpropanoid derivatives [91].

Recent studies on raw extracts of the bark of *S. brasilienisis* have revealed its anti-inflammatory and antialgic effects. These effects appear to be due to the inhibition of central and peripheral pain transmission. Due to their mechanism of action, they inhibit the TNF-α proinflammatory factor by reducing the spread of inflammatory processes so that they neutralise reactive oxygen species, which also interfere with the mechanism of pain transmission. These effects of the extract are mainly attributed to gallic acid which is also a chemical marker of the species [16].

## 5. Conclusions

The bark of woody vascular plants is often considered a forest waste, but it can be an important source of bioactive compounds with a high potential for capitalisation. The large number of publications regarding the analysis of phenolic compounds extracted from the bark of woody vascular plants is testament to their importance and their value. Thus, many studies have focussed on optimising extraction methods and the identification of bioactive compounds. Numerous global extracts obtained from the bark of plants can have important biological effects such as antioxidant, antibacterial, anti-inflammatory, antitumoral, etc. Future research directions should be directed towards identification and isolation of bioactive compounds and the description of the mechanism of action of these compounds in living systems. Consequently, biologically active compounds obtained from the bark of woody plants could be exploited on an industrial scale.

## Figures and Tables

**Table 1:** Factors involved in different extraction methods according to the studied species.

Source of Bark: Scientific Name (Family)–Commun Name	Extraction	Solvent	Time (min)	Temperature °C	Reference
*Abies alba* Mill (Pinaceae)–silver fir	CWBE	ethyl acetate	120	70	[53]
*Acacia confuse* Merr. (Fabaceae)	CWBE	NaOH 1%	60	100	[54]
*Acacia cornigera* (L.) Willd. (Fabaceae), bullhorn acacia	CWBE	petroleum ether, chloroform, methanol	4320	RT	[12]
*Acacia ferruginea* DC. (Fabaceae)–rusty acacia	SE	methanol 70%	-	-	[55]
*Acacia mearnsii* Wild. (Fabaceae) *(Acacia mollissima)*–black wattle	MAE	methanol:water 80:20	-	-	[31]
*Acacia nilotica* L. (Fabaceae)–gum arabic tree	CWBE	methanol:ethanol, acetone:water	480	RT	[29]
*Acanthopanax leucorrhizus* (Oliv.) Harms (Araliaceae)	ME	ethanol 90%	1440	RT	[44]
*Acer saccharum* Marshal (Sapindaceae)–sugar maple	ME	ethanol 95%	-	-	[56]
*Allophylus africanus* Beauv. (Sapindaceae)	CE	water	30	-	[45]
*Anacardium occidentale* L. (Anacardiaceae)–cashew tree	ME	water	120	RT	[57]
*Anogeissus leiocarpa* DC. (Combretaceae)–African birch	SE	ethanol	300	-	[58]
*Azadirachta indica* A.Juss. (Meliaceae)–nimtree or Indian lilac	CWBE	methanol:ethanol:acetone:water	480	RT	[29]
*Betula alleghaniensis* Britt. (Betulaceae)–yellow birch or golden birch	UAE	ethanol 95%	-	-	[56]
*Betula papyrifera* Marshall (Betulaceae)–Paper birch	UAE	ethanol:water 80:20	180	50	[35]
*Byrsonima crassifolia* (L.) Kunth (Malpighiaceae)–golden spoon	ME	petroleum ether:chloroform:methanol	4320	RT	[12]
*Caraipa densifolia* Mart. (Calophyllaceae)	SE	hexane:methanol	180	-	[59]
*Cassia auriculata* (L.) Roxb. (Fabaceae)–matura tea tree	UAE	water	300	-	[60]
*Castanea sativa* Mill. (Fagaceae)–sweet chestnut	UAE	methanol	30	RT	[18]
	SE	*n*-hexane, acetone, ethanol, methanol	900	-	[32]
*Cayratia pedata* Lam. (Vitaceae)	UAE	methanol	10	RT	[61]
*Chloroxylon swietenia* DC. (Rutaceae)–East Indian satinwood or buruta	UAE	methanol	10	RT	[61]
*Cinnamon* sp. (Lauraceae)	CWBE	ethyl acetate	600	30	[62]
*Coutarea hexandra* (Jacq.) K. Schum (Rubiaceae)	ME	ethanol 95%	10,080	-	[63]
*Diotacanthus albiflorus* Benth. (Acanthaceae)	UAE	methanol	10	RT	[61]
*Drypetes klainei* Pierre ex Pax (Putranjivaceae)	ME	water	180	RT	[15]
*Erythrina suberosa* Roxb. (Fabaceae)–Corky coral tree	CWBE	methanol	1080	RT	[64]
*Eucalyptus camaldulensis* Dehnh. (Myrtaceae)	UAE	ethanol	-	40–50	[36]
*Eucalyptus globulus* Labill (Myrtaceae)–Tasmanian bluegum, blue gum	SE	*n*-hexane, acetone, ethanol, methanol	900	-	[32]
	CWBE	ethanol:water 80:20 (*v*/*v*)	360	82.5	[65]
*Eucalyptus grandis* W.Hill ex. Maiden (Myrtaceae)–rose gum	SE	dichloromethane	360	-	[47]
*Eucalyptus maidenii* F. Muell (Myrtaceae)–Maiden’s Gum	SE	dichloromethane	360	-	[47]
*Eucalyptus sideroxylon* A.Cunn. (Myrtaceae)–mugga, red ironbark	UAE	ethanol:water	60	50	[66]
*Eucalyptus urograndis* (Myrtaceae)–Hybrid *E.grandis* x *E. urophylla*	SE	dichloromethane	360	-	[47]
*Euclea undulata* Thunb. (Ebenaceae)–small-leaved guarri, common guarri	CWBE	acetone	4320	RT	[67]
*Eucommia ulmoides* Oliv. (Eucommiaceae)	MAE + UAE	water, ethanol	10–60	20–60	[46]
*Eugenia jambolana* Lam. (Myrtaceae)–Jamun, black plum	CWBE	methanol 80%, ethanol 80%, acetone:water 80:20	480	RT	[29]
*Fagus sylvatica* L. (Fagaceae)–common beech	CWBE	water, methanol:water 80:20, ethanol:water 80:20	120, 300, 1440	RT	[25]
	MAE	water, methanol:water and ethanol:water (80:20)	10, 20	60, 80, 100, 120	
	UAE	water, methanol:water 80:20, ethanol:water 80:20	10, 20, 30	RT	
*Ficus talboti* King. (Moraceae)–talbot fig	SE	methanol	-	-	[68]
*Flourensia cernua* DC. (Asteraceae)–American tarwort and tarbush	UAE	ethanol	-	40–50	[36]
*Guazuma ulmifolia* Lam. (Malvaceae)–West Indian elm or bay cedar	CWBE	petroleum ether, chloroform, methanol	4320	RT	[69]
*Goniothalamus velutinus* Airy Shaw (Annonaceae)	SE	absolute methanol	600	-	[70]
*Hugonia mystax* Cav. (Linaceae)	CWBE	distilled water, methanol ethanol	-	RT	[13]
*Hymenaea courbaril* L. (Fabaceae)	SFE	CO_2_ and water (9:1, *v*/*v*)	-	56.85	[42]
*Jatropha dioica* Sesse (Euphorbiaceae)–leatherstem	UAE	ethanol	-	40–50	[36]
*Lafoensia pacari* A. St.-Hil (Lythraceae)	ME	absolute ethanol	10,080	RT	[20]
*Larix laricina* K. Koch (Pinaceae)–tamarack or American larch	CWBE	ethanol 80%	-	-	[71]
*Liriodendron tulipifera* L. (Magnoliaceae)–tulip tree, American tulip tree, tulipwood	CWBE	oxalic acid 0.1 M	60	170	[72]
*Malus domestica* Miller (Rosaceae)–apple tree	CWBE MAE	ethanol:water, 1:4 ethanol:water, 60:40 *v*/*v*	120 20	55 100	[22]
*Morus alba* L. (Moraceae)–white mulberry	CWBE	methanol:water	-	-	[73]
*Picea abies* L. (Pinaceae)–european spruce	UAE	ethanol:water 50%, 70% (*v*/*v*)	30–60	40–60	[48]
	CWBE	water	10–120	60–90	[19]
	CWBE	distilled water	120	90	[50]
	UAE	ethanol:water 70% (*v*/*v*)	5–75	45–60	[49]
*Picea mariana* (Mill.) Britton (Pinaceae)–the black spruce	SE	water	60	-	[11]
*Pinus sylvestris* L. (Pinaceae)–Scots pine	CWBE	acetone:water	3 × 5	100	[74]
*Pinus brutia* Tenore (Pinaceae)–Turkish pine	CWBE	distilled water	60	70	[75]
*Pinus pinaster* Aiton (Pinaceae)–the maritime pine, cluster pine	MAE	ethanol:water 80:20	30	-	[38]
	CWBE	distilled water, ethanol, methanol	-	-	[76]
	CWBE	water:NaOH:Na_2_SO_3_:NaHSO_3_	120	70–80	[77]
*Pinus radiata* D.Don (Pinaceae)–Monterey pine, insignis pine or radiata pine	CWBE	Water	60	100	[78]
	CWBE	deionized water	30	95–99	[79]
	CWBE	ethanol:water, 3:1 (*v*/*v*)	120	120	[10]
*Populus nigra* L. (Salicaceae)–black poplar	UAE ME	ethanol:water 70:30 ethanol:water 70:30	60 -	- RT	[27]
*Punica granatum* L. (Lythraceae)–Pomegranate	CWBE	methanol	-	RT	[80]
*Prunus domestica* L. (Rosaceae)–Plums	UAE	7 ethanol and HCl 1%, 2,6-di-tety-butyl-4-methylphenol (BHT)	-	-	[81]
*Quercus robur* L. (Fagaceae)–common oak, pedunculate oak	MAE	hydroalcoholic solution of methanol and ethanol	5–120	100	[82]
*Rhus verniciflua* (Stokes) F.Barkley (Anacardiaceae)–Chinese lacquer tree	SE	ethanol	-	-	[83]
*Salix alba* L. (Salicaceae)–white willow	ME	ethanol:water 70:30	1440	-	[84]
*Saraca asoca* (Roxb.) Willd (Fabaceae)–the ashoka tree	CWBE	methanol	1440	RT	[85]
*Schinopsis brasiliensis* Engl. (Anacardiaceae)–baraúna	ME	ethanol 90%	7200	RT	[47]
*Sclerocarya birrea* (A. Rich.) Hochst. (Anacardiaceae)–marula	ME	distilled water	2880	RT	[86]
*Shorea roxburghii* D.Don (Dipterocarpaceae)	CWBE	acetone:methanol	-	-	[87]
*Strychnos minor* Dennst. (Loganiaceae)	UAE	methanol	10	RT	[61]
*Strychnos nux-vomica* Dennst. (Loganiaceae)–the strychnine tree, nux vomica, poison nut	UAE	methanol	10	RT	
*Sweetia panamensis* Yakovlev (Fabaceae)	CWBE	petroleum ether:chloroform:methanol	4320	RT	[12]
*Terminalia brownie* Fresen (Combretaceae)	SE	absolute ethanol	300	-	[58]
*Terminalia arjuna* Wight & Arn (Combretaceae)–arjun tree	SE	petroleum ether:ethanol	-	60–80	[88]
	MAE	distilled water	5	-	[89]
	ME	ethanol	7200	-	[90]
	CWBE	methanol:ethanol:acetone:water	480	RT	[29]
*Terminalia laxiflora* Engl. & Diels (Combretaceae)	SE	absolute ethanol	300	-	[58]
*Trichilia catigua* A.Juss. (Meliaceae)	CWBE	distilled water	20	100	[91]
*Turnera diffusa* Willd (Passifloraceae)–Damiana	UAE	ethanol	-	40–50	[36]
*Ulmus pumila* L. (Ulmaceae)–the Siberian elm	EAE	cellulose, pectinase, β-glucosidase	60–90	40–60	[51]
	UAE	ethanol 50%	10–90	52	
	CWBE	ethanol 50%	10–90	52	
*Vitex doniana* L. (Lamiaceae)–Black plum	ME	distilled water	2880	RT	[92]
*Ziziphus jujuba* Mill. (Rhamnaceae)–Jujube	CWBE	ethanol, methanol, hexane, acetone	160	70	[39]
	UAE	methanol	20–60	RT	
	SE	methanol	40–140	68	
	MAE	methanol	4	-	

RT—room temperature, CWBE—classic water bath extraction, ME—extraction by maceration, SE—Soxhlet extraction, UAE—ultrasound-assisted extraction, MAE—microwave-assisted extraction, SFE—supercritical fluid extraction, EAE—enzymatic assisted extraction.

**Table 2:** The biological action of the extracts obtained from the bark of woody vascular plants.

Source of Bark: Scientific Name (Family)–Commun Name	Composition of Extract	Action/Application	Reference
*Acacia cornigera* (L.) Willd. (Fabaceae), bullhorn acacia	-	anti-inflammatory topical	[12]
*Allophylus africanus* Beauv. (Sapindaceae)	Apigenin, Luteolin, Vitexin, Apigetrin, Cymaroside	anti-inflammatory	[45]
*Anogeissus leiocarpa* DC. (Combretaceae)–African birch	Gallic acid, ellagitannin, Ampelopsin, Gallotannin, Epigallocatechin gallate, Ellagic acid derivative	antibacterial	[58]
*Byrsonima crassifolia* (L.) Kunth (Malpighiaceae)–golden spoon	-	anti-inflammatory topical	[12]
*Caraipa densifolia* Mart. (Calophyllaceae)	procaynidin dimer B2, procyanidin trimer C1, epicatechin, lupeol, betulinic acid	cancer prevention chemoprevention	[59]
*Cayratia pedata* Lam. (Vitaceae)	quercetin, *o*-coumaric acid, gallic acid	Antioxidant antiviral, cytotoxic	[61]
*Chloroxylon swietenia* DC. (Rutaceae)–East Indian satinwood or buruta	quercetin, ferulic acid, gallic acid	Antioxidant antiviral, cytotoxic	
*Diotacanthus albiflorus* Benth. (Acanthaceae)	quercetin, *o*-coumaric acid, ferulic acid, gallic acid	Antioxidant antiviral, cytotoxic	
*Erythrina suberosa* Roxb. (Fabaceae)–Corky coral tree	α-Hydroxyerysotrine, 4′-Methoxy licoflavanone (MLF), Alpinumisoflavone, (AIF), Wighteone	antitumoral, cytotoxic effect on HL-60 cells	[64]
*Eucalyptus grandis* W.Hill ex. Maiden (Myrtaceae)–rose gum	quinic acid, gallic acid, protocatechuic acid, catechin, ellagic acid, ellagic, acid-rhamnoside	antioxidant	[47]
*Eucalyptus maidenii* F. Muell (Myrtaceae)–Maiden’s Gum	quinic acid, gallic acid, protocatechuic acid, catechin, chlorogenic acid, ellagic acid, taxifolin, quercetin, mearnsetin, naringenin, ellagic acid-rhamnoside	antioxidant	
*Eucalyptus sideroxylon* A.Cunn. (Myrtaceae)–mugga, red ironbark	Monosaccharides, glucose, xylose, galactose, arabinose, mannose, rhamnose	antioxidant	[66]
*Eucalyptus urograndis* (Myrtaceae)–Hybrid *E.grandis* x *E. urophylla*	quinic acid, gallic acid, protocatechuic acid, catechin, ellagic acid, ellagic, acid-rhamnoside	antioxidant	[47]
*Fagus sylvatica* L. (Fagaceae)–common beech	Procyanidin, Epicatechin, Coumaric acid, Coniferin, Quercetin, Taxifolin-*O*-hexoside, Coumaric, acid-di-*O*-hexoside, Syringic acid-di-*O*-hexoside, Coniferyl alcohol-*O*-hexoside-*O*-pentoside	antioxidant	[26]
*Ficus racemosa* L. (Moraceae)–cluster fig tree, Indian fig tree or goolar (gular)	Kaempferol, Quercetin, Naringenin, Baicalein	normalizes glycogenol levels and hepatic glycogen, normalizes blood glucose levels	[17]
	-	Antioxidant renoprotective activity	[103]
*Ficus talboti* King. (Moraceae)–talbot fig	Gallic acid, Caffeic acid, Rutin, Ellagic acid, Quercetin, Kaempferol	hypocolesterolemiant, antidiabetic—increases the insulin sensitivity of pancreatic β cells, normalizes blood glucose level, antioxidant	[68]
*Guazuma ulmifolia* Lam. (Malvaceae)–West Indian elm or bay cedar	Flavanocoumarin epiphyllocoumarin, Epiphyllocoumarin-[4β→8]-(−)-epicatechin	anti-inflammatory, antioxidant	[69]
*Hugonia mystax* Cav. (Linaceae)	Gallic acid, catechol, caffeic acid, vanillin, *p*-coumaric acid, ferulic acid	anti-inflammatory, antioxidant, antirheumatic	[13]
*Larix laricina* K. Koch (Pinaceae)–tamarack or American larch	Rhaponticin, Rhapontigenin, Piceatannol, Taxifolin	antioxidant	[71]
*Lafoensia pacari* A. St.-Hil (Lythraceae)	Ellagic acid	anti-ulcerative-gastric hypopoietic, gastroprotector effect	[20]
*Liriodendron tulipifera* L. (Magnoliaceae)–tulip tree, American tulip tree, tulipwood	Furan-2-carboxylic acid, Mannose, β-d-glucopyranose, 3,5-dimethoxyphenol, 3,4-dimethoxy-mandelic acid, 2-Amino-3-hydroxybenzoic acid	antioxidant	[72]
*Malus domestica* Miller (Rosaceae)–apple tree	Gallic acid, Chlorogenic acid, Vanillic acid, Caffeic acid, Syringic acid, Ferulic acid, Sinapic acid, Resveratrol, Myricetin, Quercetin, Cinnamic Acid	antioxidant in food, cosmetics and pharmaceutical industry	[12]
*Picea mariana* (Mill.) Britton (Pinaceae)–the black spruce	Neolignans, Lignans: pinoresinol, Secoisolariciresinol, isolariciresinol, Epi-pinoresinol. Phenolic acids: *trans*-*p*-coumaric acid, vanillic acid, protocatechuic acid. Stilbenes: transresveratrol. Flavonoids: Kaempferol, quercetin, taxifolin, epitaxifolin, pallasiin, mearnsetin. Other phenolic compounds: *p*-vanillin, dihydroconiferyl alcohol	antiproliferative, antioxidant, anti-inflammatory	[11]
*Pinus radiata* D.Don (Pinaceae)–Monterey pine, insignis pine or radiata pine	Dihydroxybenzoic acid, 3,4-Dihydroxyphenylacetic acid, *p*-Hydroxybenzoic acid, Proanthocyanidin B2, Catechin, Epicatechin, Syringic acid, Taxifolin, Quercetin, Homovanillic acid, Epigallocatechin	antioxidant	[10]
*Rhamnus alaternus* L. (Rhamnaceae)–Italian buckthorn	Emodin, Chrysophanol, Physcion	Antioxidant antimicrobial	[104]
*Schinopsis brasiliensis* Engl. (Anacardiaceae)–baraúna	Gallic acid	Analgesic anti-inflammatory topical	[28]
*Solidago canadensis* L. (Asteraceae)–Canada goldenrod	-	Antioxidant antimicrobial	[94]
*Strychnos minor* Dennst. (Loganiaceae)	quercetin, coumaric acid, ferulic acid, gallic acid	antioxidant, antiviral, cytotoxic	[61]
*Strychnos nux-vomica* Dennst. (Loganiaceae)–the strychnine tree, nux vomica, poison nut	quercetin, ferulic acid, gallic acid	antioxidant, antiviral, cytotoxic	
*Sweetia panamensis* Yakovlev (Fabaceae)	-	anti-inflammatory topic	[12]
*Terminalia arjuna* Wight & Arn (Combretaceae)–arjun tree	-	Antioxidant antimutagenic	[88]
*Terminalia brownie* Fresen (Combretaceae)	Gallic acid, Ellagitannin, Punicalagin, Gallotannin, Corilagin	antibacterial	[58]
*Terminalia laxiflora* Engl. & Diels (Combretaceae)	Gallic acid, EllagitanninEllagic acid glucuronide, GallotanninMethylellagic acid glucuronide, Methyl-(*S*)-flavogallonate and its isomer	antibacterial	
*Terminalia tomentosa* Wight & Arn (Combretaceae)–Asan, Indian Laurel, Silver grey wood	-	anti-inflammatory	[105]
*Trichilia catigua* A.Juss. (Meliaceae)	Catechin, Procyanidin, Epicatechin, Apocynin E, Cinchonain I, 3-Methoxybenzoylquinic acid	antioxidant, anti-inflammatory, antidepressant, neuroprotective	[91]
